# Supplementary material for: End Sequence Analysis Toolkit (ESAT) expands the extractable information from single-cell RNA-seq data
Source: Genome Res. 2016 Oct;26(10):1397–410. doi: 10.1101/gr.207902.116 (PMC5052061; doi:10.1101/gr.207902.116)
Supplement: Supplemental Material [file supp_gr.207902.116_Supp_Materials.tar.gz › ESAT-esat_v0.1/doc/html/classbroad_1_1core_1_1motif_1_1_p_w_m_utils.html]

ESAT: broad.core.motif.PWMUtils Class Reference


ESAT


- Main Page
- Related Pages
- Packages
- Classes
- Files

- Class List
- Class Index
- Class Hierarchy
- Class Members

All Classes Namespaces Files Functions Variables Enumerator Pages

- broad
- core
- motif
- PWMUtils

Static Public Member Functions |
Static Public Attributes |
List of all members

broad.core.motif.PWMUtils Class Reference

Collaboration diagram for broad.core.motif.PWMUtils:

[legend]

|  |  |
| --- | --- |
| Static Public Member Functions | |
| static void | main (String[] args) throws IllegalArgumentException, Exception |
|  | |
| static double[] | computeSequenceBGSequences (Map< String, Sequence > chrsByName, AnnotationReader<?extends GenomicAnnotation > reader) throws IOException |
|  | |
| static Map  < PositionWeightMatrix,   LightweightGenomicAnnotation > | scanRegion (int shuffles, String prefix, double minScoreToWrite, boolean maxOnly, String outdir, List< PositionWeightMatrix > pwms, String chr, Annotation annot, double[] bgNucleotideFreqs, short[] encChrSeq) throws IOException |
|  | |

|  |  |
| --- | --- |
| Static Public Attributes | |
| static final String | USAGE |
|  | |

## Member Function Documentation

|  |  |  |  |
| --- | --- | --- | --- |
| static double [] broad.core.motif.PWMUtils.computeSequenceBGSequences | ( | Map< String, Sequence > | *chrsByName*, |
|  |  | AnnotationReader<?extends GenomicAnnotation > | *reader* |
|  | ) |  | throws IOException |

static

Here is the call graph for this function:

|  |  |  |  |  |  |
| --- | --- | --- | --- | --- | --- |
| static void broad.core.motif.PWMUtils.main | ( | String[] | *args* | ) | throws IllegalArgumentException, Exception |

static

Here is the call graph for this function:

|  |  |  |  |
| --- | --- | --- | --- |
| static Map<PositionWeightMatrix, LightweightGenomicAnnotation > broad.core.motif.PWMUtils.scanRegion | ( | int | *shuffles*, |
|  |  | String | *prefix*, |
|  |  | double | *minScoreToWrite*, |
|  |  | boolean | *maxOnly*, |
|  |  | String | *outdir*, |
|  |  | List< PositionWeightMatrix > | *pwms*, |
|  |  | String | *chr*, |
|  |  | Annotation | *annot*, |
|  |  | double[] | *bgNucleotideFreqs*, |
|  |  | short[] | *encChrSeq* |
|  | ) |  | throws IOException |

static

Here is the call graph for this function:

Here is the caller graph for this function:

## Member Data Documentation

final String broad.core.motif.PWMUtils.USAGE

static

**Initial value:**

= "Usage: ArrayDesignUtilities TASK=<task\_num> <task\_args>\n" +

"\tTasks:\n" +

"\t\t1. Compute log likelohood score thresholds for a given PWM set. This task will compute the distribution of scores then output thresholds for commonly used quantiles. -pwm <File containing one or more PWM matrices> -pA <new equilibrium probability for A> -pC <new equilibrium probability for C> -pG <new equilibrium probability for G> -pT <new equilibrium probability for T> -out <file name for score quantile cutoff per pwm file or standard out if none is specified> -outdir <output directory or current directory if this parameter is not specified> [-prefix <A prefix for each pwm distribution file>]> " +

"\n\t\tscan. Scan a set a set of genomic regions for PWM ocurrencens -pwm <File containing one or more PWM matrices> -regions <Genomic Annotations> -seqdir <Sequence directory> -maxOnly <If only the maximal hit per region should be reported> -outdir <Output directory to write output file> [-minScoreToWrite <Filter output by minimum score, reudces output file size> -prefix <Prefix to use for each PWM hit file> -shuffles <Number of pwm shuffles to asses significance>]"+

"\n\t\tpermute. Permute a PWM matrix -pwm <File contining one or more PWM matrics> -out <Output file with permuted matrices or standard out in not specified>"+

"\n\t\treverse. Reverse-compliment a PWM -pwm <File contining one or more PWM matrics> -out <Output file with permuted matrices or standard out in not specified>" +

"\n\t\tscanGenome. Scan the genome for PWM ocurrencens -pwm <File containing one or more PWM matrices> -seqdir <Sequence directory> -outdir <Output directory to write output file> -minScoreToWrite <Filter output by minimum score, reudces output file size> [-prefix <Prefix to use for each PWM hit file> -shuffles <Number of pwm shuffles to asses significance>]"+

"\n"

---

The documentation for this class was generated from the following file:

- java/broad/core/motif/PWMUtils.java


---

Generated on Fri Aug 15 2014 15:10:54 for ESAT by  

 1.8.7
